# Supplementary material for: Probing magnon–magnon coupling in exchange coupled Y3Fe5O12/Permalloy bilayers with magneto-optical effects
Source: Sci Rep. 2020 Jul 28;10:12548. doi: 10.1038/s41598-020-69364-6 (PMC7387351; doi:10.1038/s41598-020-69364-6)
Supplement: Supplementary file 1 — Supplementary Information 1. [file 41598_2020_69364_MOESM1_ESM.pdf]

Supplemental Materials:

Probing magnon-magnon coupling in exchange coupled $Y_3Fe_5O_{12}$/Permalloy bilayers with magneto-optical effects

Yuzan Xiong, Yi Li, Mouhamad Hammami, Rao Bidthanapally, Joseph Sklenar, Xufeng Zhang, Hongwei Qu, Gopalan Srinivasan, John Pearson, Axel Hoffmann, Valentine Novosad, and Wei Zhang.

1. Measurement setup:

The magnetization dynamics are detected optically by the magneto-optical Kerr and Faraday effects for Py and YIG, respectively, by measuring the out-of-plane component of precessing magnetization and using one single light wavelength at 1550-nm. This method is advantageous in the context of studying magnon-magnon coupling of hybrid insulator-metal systems, since it avoids the complication of using multiple wavelengths in the visible range for detecting respective metals and insulators. In our system, the optical power used for the 1550-nm laser is between 6 - 8 mW and we detect a modulated voltage around 50 - 200 μV (specific values vary between different samples and usage of additional microwave amplifiers, etc). A heterodyne method is adopted to enable precessional phase extraction using a setup illustrated in Fig.S-1. A single microwave source was used to simultaneously modulate the detecting laser light (optical path), and drive the FMR of the sample (electrical path) with a coplanar waveguide (CPW). The laser light was modulated at the microwave source frequency using an electro-optic intensity modulator. The modulated laser light can be polarized by either a fiber polarizer (used in combination with a polarization controller) or a free-space thin-film polarizer, before being focused onto the sample surface. We use an optical tap ($\sim$ 10%), a GRIN lens, and another free-space polarizer to monitor the polarization during the whole measurements. The focused light spot is set to $\sim$ 40 μm in this work. A microwave diode was used simultaneously to measure the inductive FMR absorption through the CPW.

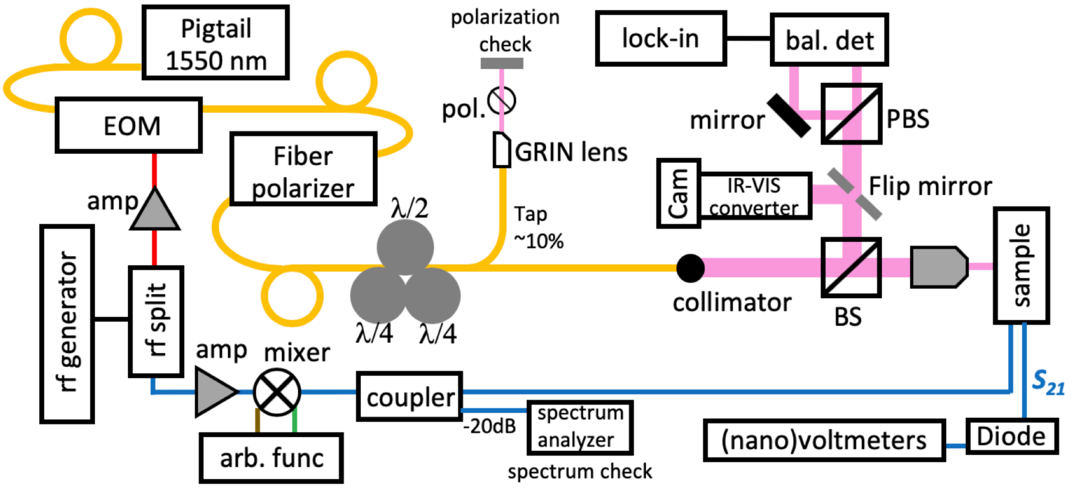

FIG. S-1. Schematics of the measurement setup. After the rf splitter, the optical path (upper part) contains amplifier, 1550 nm infrared laser module, electro-optic modulator (EOM), polarizer, polarization controller, beam splitter (BS) and focusing lens; the electrical path (lower part) contains amplifier, mixer, coupler, spectrum analyzer, diode, and nanovoltmeter. (PBS = polarizing beam splitter, Cam = camera, bal.det = balancing detector, arb. func = arbitrary waveform generator.)

For a heterodyne detection, the microwave signal along the electrical path was IQ-mixed with a low-frequency (100 kHz) signal provided by a waveform generator. The voltage amplitude, offset, and phase for the respective "I" and "Q" channels were optimized to ensure the power of the upper side-band of the microwave signal (which was subsequently used for FMR excitation) far exceeds those of the central and lower side-band (>20 dB). We use a directional coupler (−20 dB), and a real-time spectrum analyzer to monitor the central and side-bands throughout the whole measurement. The resultant, out-of-plane, dynamical Kerr and Faraday responses of the sample were then probed by the modulated light, sent into a balancing detector after polarization splitting, and analyzed by a lock-in amplifer. Avoiding the use of a VNA offers more flexibility in configuring the setup

for different dynamic measurements [1, 2]. The use of fiber-optical components also increases the robustness against external vibrations as well as reduces the cost as compared with conventional VNA experiments [3–7].

The measured signal is described with the optical and electrical phases and delays, following: $X \propto m_z P_0 \cos(\phi_{\rm L} - \phi_{\rm m})$ and $Y \propto -m_z P_0 \sin(\phi_{\rm L} - \phi_{\rm m})$, where m_z is the z-component of the dynamic magnetization (for YIG or Py), P_0 is the laser light intensity, $\phi_{\rm L}$ is the phase accumulated due to the optical path length, and $\phi_{\rm m}$ is the magnetization phase, which includes contributions due to $\phi_{\rm MW}$ (the microwave path), $\phi_{\rm h}$ (possible phase delay between waveguide current and effective driving field, $h_{\rm rf}$), and ϕ_χ (the phase of the magnetic response to the field) [1]. The $\phi_{\rm L}$ and $\phi_{\rm MW}$ are dependent on the optical and electrical paths, respectively, and the resulted path difference can be tuned by the fiber and microwave-cable lengths or using a microwave phase shifter [2]. It is noted that the fiber-optic system makes it particularly convenient to engineer the path difference by simply adding optical fibers at desirable lengths. The total amplitude, as calculated by $\sqrt{X^2 + Y^2}$, resembles the conventional microwave diode or the VNA measurements (S_{21}) [3–6].

In our measurements, the optical signals should have negligible effect from $\phi_{\rm L}$, $\phi_{\rm MW}$, and $\phi_{\rm h}$, which are solely geometrical. The YIG FMR from the Faraday effect is detected as: $X_{\rm YIG} \propto m_{\rm z(YIG)} P_0 \cos[\phi_{\rm L} - \phi_{\rm MW} - \phi_{\rm h} - \phi_{\chi({\rm YIG})}]$, and $Y_{\rm YIG} \propto -m_{\rm z(YIG)} P_0 \sin[\phi_{\rm L} - \phi_{\rm MW} - \phi_{\rm h} - \phi_{\chi({\rm YIG})}]$. The Py FMR, on the other hand, is due to the Kerr effect, detected as: $X_{\rm Py} \propto m_{\rm z(Py)} P_0 \cos[\phi_{\rm L} - \phi_{\rm MW} - \phi_{\rm h} - \phi_{\chi({\rm Py})}]$, and $Y_{\rm Py} \propto -m_{\rm z(Py)} P_0 \sin[\phi_{\rm L} - \phi_{\rm MW} - \phi_{\rm h} - \phi_{\chi({\rm Py})}]$. The strong magnon-magnon coupling is also implied from the detecting mechanism of the PSSWs: due to the standing-wave nature, the Faraday optical response cancels out inside the YIG bulk except at the boundaries. The observed PSSW signals are thus dominated by the Py-YIG hybrid Kerr resonance at the interface, influenced by the magnon-magnon coupling with the bulk YIG PSSW modes.

2. Dataset for the 10-nm-Py sample:

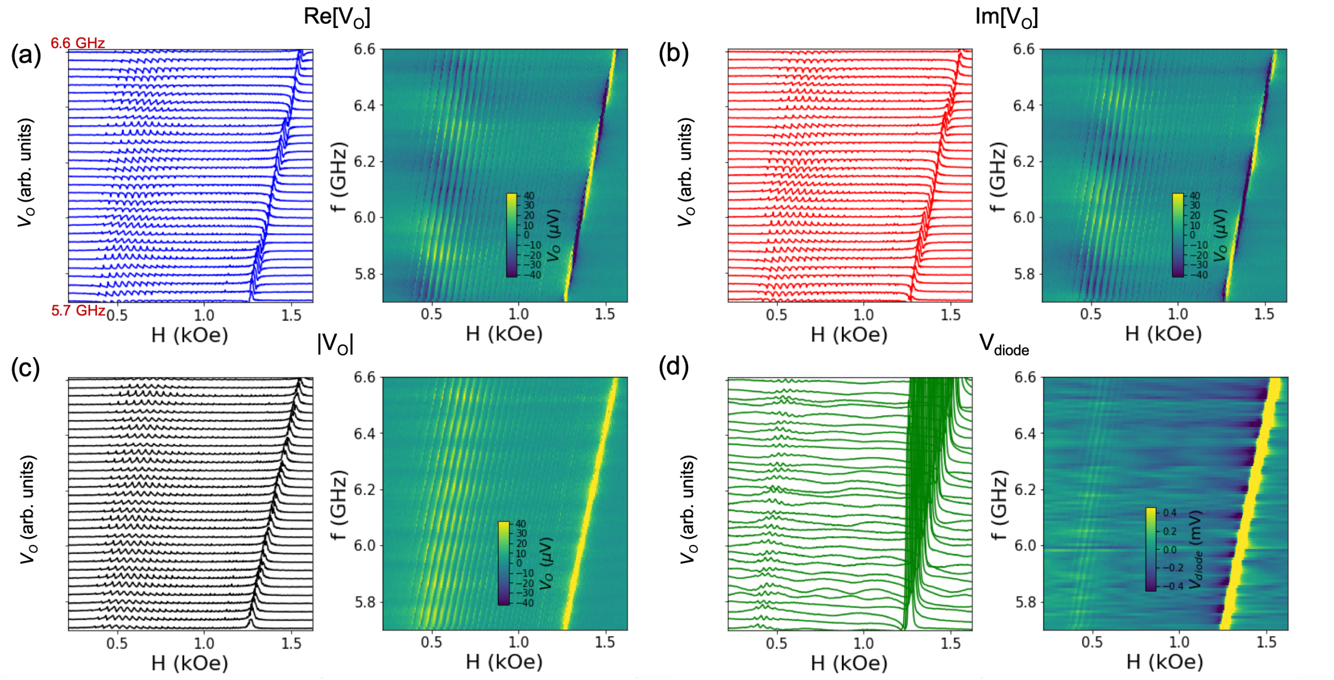

FIG. S-2. Dataset for the 10-nm-Py sample. The optically detected (a) in-phase, Re[$V_{\rm O}$], (b) quadrature, Im[$V_{\rm O}$], (c) total optical signal, $V_{\rm O}$, and (d) microwave diode signal, $V_{\rm diode}$, as a function of the magnetic field and frequency (5.7 - 6.6 GHz).

The complete fine-scan lineshape and dispersion data for the 10-nm-Py sample are summarized in Fig.S-2. A total of more than 40 PSSW modes can be identified at a broad range of frequencies. The frequency step used in the fine scan is 0.01 GHz. By comparing the total optical signal, V_O in Fig.S-2(c), with the simultaneous electrical diode signal, $V_{\rm diode}$ in Fig.S-2(d), we found that more PSSW modes can be identified from the optical dataset than the electrical counterpart. In addition, the optical dataset has a much less noisy background. Such a direction comparison further shows the advantage of using magneto-optical effects for the present and relevant studies.

3. Dataset for the 30-nm-Py sample:

To supplement with the Fig. 2 in the main text, The full- and fine-scan dispersion data, as well as the corresponding theoretical modeling data, for the 30-nm-Py sample are summarized in Fig.S-3, showing the respective in-phase, Re[V_O], and quadrature, Im[V_O] signals.

Clear phase evolution can be observed in the optical signals Re[V_O] and Im[V_O]. The periods of the phase evolution are the same for both YIG and Py lines, due to the fixed path difference of the measurement geometry, i.e. $\phi_L - \phi_{MW} - \phi_h$. The Py and YIG FMR lines also exhibit a small, but fixed phase offset, which could be due to either the intrinsic phase buildup from the Py-Kerr and YIG-Faraday effects, or a phase lag of the Py FMR with respect to the YIG FMR, caused by the coupled PSSWs' "dragging" the hybrid YIG-Py resonances, i.e. from a finite $\phi_{\chi(\rm Py)} - \phi_{\chi(\rm YIG)}$. It is noted that this effect is unlikely due to the additional travelling distance inside the 3-μm YIG bulk, as this thickness is too small compared with the microwave wavelength.

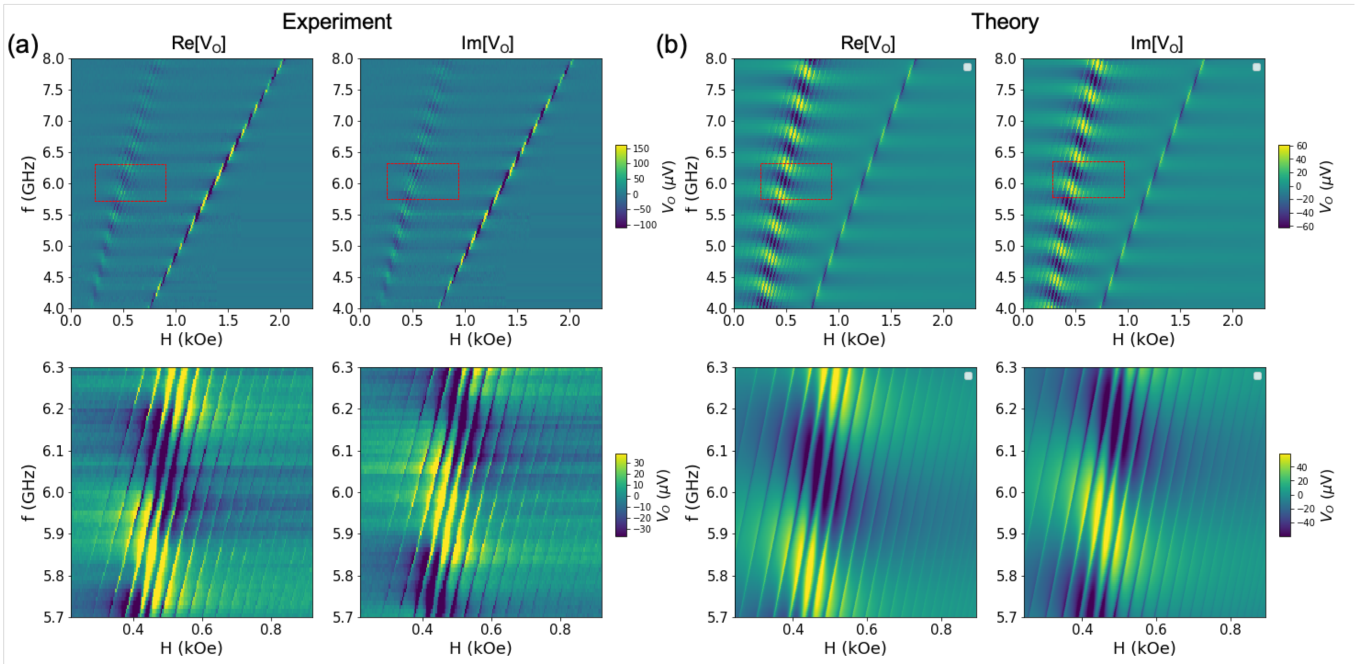

FIG. S-3. Dataset for the 30-nm-Py sample. (a) The optically detected in-phase, Re[V_O], and quadrature, Im[V_O] for the 30-nm-Py sample as a function of the magnetic field and frequency, showing the frequency-dependent phase evolution. (b) Theoretical modeling of the experimental data in (a) using the extracted fitting parameters. The bottom panels are the fine-scans at smaller field and frequency steps corresponding to the boxes in the top panel (5.7 - 6.3 GHz).

4. Dataset for the reference samples:

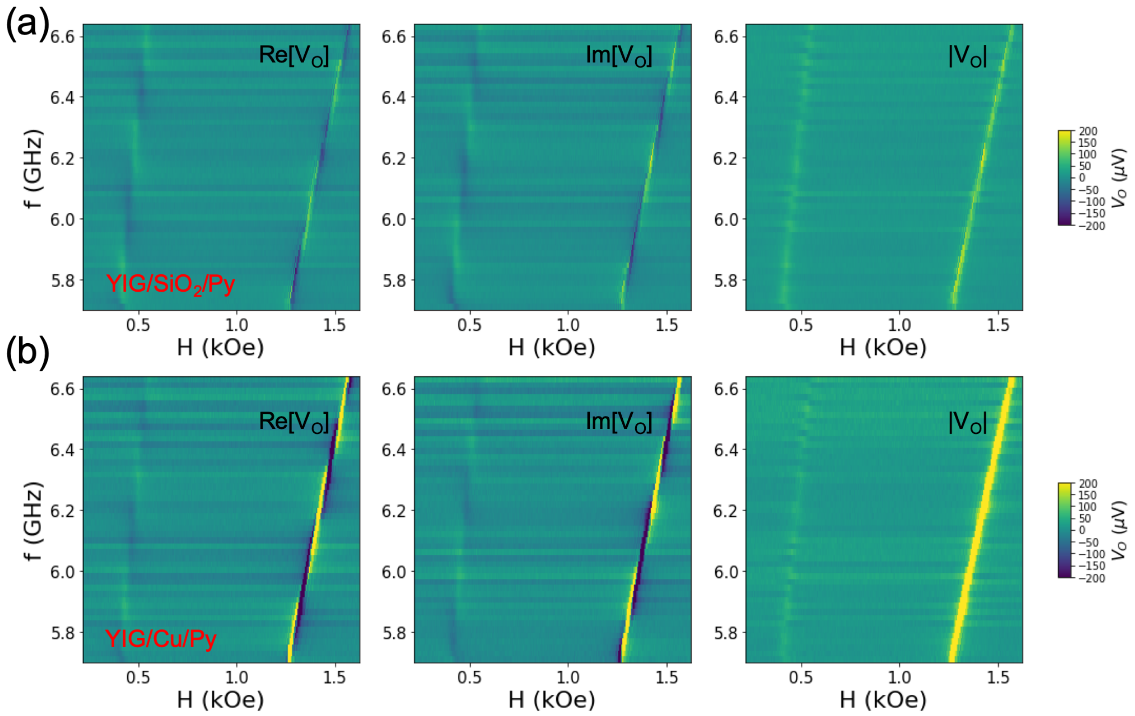

FIG. S-4. Dataset for the reference samples. The optically detected in-phase, Re[V_O], quadrature, Im[V_O], and the amplitude,V_O, for (a) YIG/SiO_2-3nm/Py10-nm and (b) YIG/Cu-3nm/Py10-nm reference samples as a function of the magnetic field and frequency.

Figure S-4 show the fine-scan optical signals, Re[V_O], Im[V_O], and the total amplitude for the reference samples, Py/SiO_2/YIG, and Py/Cu/YIG, as a function of the magnetic field and frequency. No YIG PSSWs are observed in the Py/SiO_2/YIG and Py/Cu/YIG references, suggesting that the coupling is through the exchange interaction at the interface as opposed to the dipolar interaction. In addition, the Py Kerr signal is attenuated in the Py/Cu/YIG as compared to the Py/SiO_2/YIG, which is likely due to the metal refractive index of the inserted Cu layer.

5. Separating the Py-FMR profile and the YIG-PSSWs:

To expose the pure YIG-PSSW lineshapes, we subtract, via Lorentzian fits, the Py-FMR profile from the raw signal traces, Re[V_O]. Figure S-5(a) shows the waterfall plot of the Lorentzian fits for the Re[V_O] dataset of the 10-nm-Py sample at the same frequency window as in Fig. 3(a) of the main text. The pure YIG-PSSW series after this Py-FMR subtraction is in Fig. 3(b) of the main text. In principle, such fitting can be also performed for the Im[V_O] or $|V_O|$ datasets. Figure S-5(b) shows a zoom-in example of the raw signal trace and the Py-FMR fitting at 6 GHz.

6. Phenomenological Model and linewidth enhancement:

In the presence of a uniform microwave field, the magnetization dynamics of Py, which couples to the magnetization motion of YIG via interfacial exchange, can be expressed in the field domain by the quantum optics formula [8–10] combined with the heterodyne detection mechanism [2]:

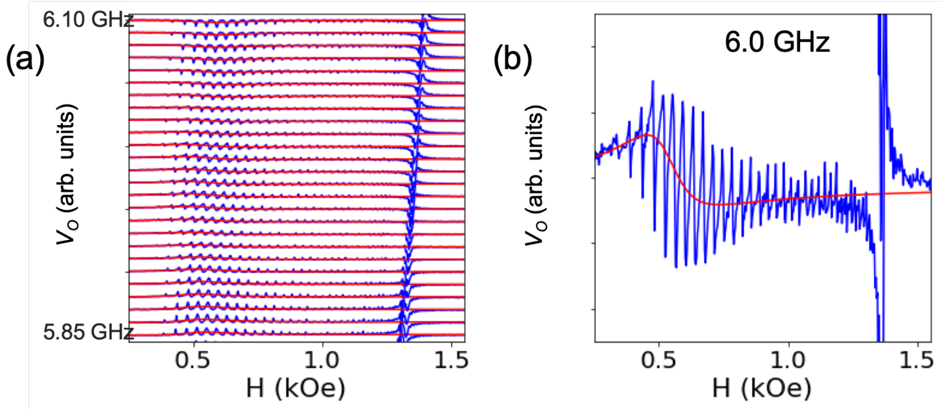

FIG. S-5. (a) Lorentzian fits for the Re[$V_{\rm O}$] dataset of the 10-nm-Py sample at the same frequency window (as in Fig. 3a of the main text). The fitting allows the subtraction of the global Py-FMR envelope and then exposure of the YIG-PSSW series (Fig. 3b of the main text). (b) A zoom-in example of the raw signal trace and fitting curve for Py-FMR at 6 GHz.

$$V_{\rm O} = \frac{Ae^{i(\phi_{\rm L}-\phi_{\rm m})}}{i(H_{\rm FMR}^{\rm Py}-H)-\Delta H_{\rm Py}+\Sigma_j \frac{g^2}{i(H_{{\rm PSSW},j}^{\rm YIG}-H)-\Delta H_{{\rm YIG},j}}} \tag{S-1}$$

This equation is used to fit the entire lineshape of the optical signals, which is proportional to $\tilde{m}_{\rm Py}$ along with the optical detection amplitude, A, and an additional phase shift caused by the measurement $\phi_{\rm L}-\phi_{\rm m}$ introduced in terms of $e^{i(\phi_{\rm L}-\phi_{\rm m})}$. In order to quantitatively correlate the enhanced YIG-linewidth with the overlapped resonance (resonance distance) of YIG and Py in the MIT regime (Fig. 3 in the main text). We further derive the formula S-1 as:

$$\begin{aligned} V_{\rm O} &= \frac{Ae^{i(\phi_{\rm L}-\phi_{\rm m})}}{i(H_{\rm FMR}^{\rm Py}-H)-\Delta H_{\rm Py}+\Sigma_j \frac{g^2}{i(H_{{\rm PSSW},j}^{\rm YIG}-H)-\Delta H_{{\rm YIG},j}}} \\ &= (-i)\frac{Ae^{i(\phi_{\rm L}-\phi_{\rm m})}}{(H-H_{\rm FMR}^{\rm Py})-i\Delta H_{\rm Py}-\Sigma_j \frac{g^2}{(H-H_{{\rm PSSW},j}^{\rm YIG})-i\Delta H_{{\rm YIG},j}}} \end{aligned} \tag{S-2}$$

In the vicinity of the MIT regime, H is close to $H_{{\rm PSSW},j}^{\rm YIG}$. To consider the lineshape evolution within the range of $\Delta H_{{\rm YIG},j}$, we can rewrite H as $H = H_{{\rm PSSW},j}^{\rm YIG}+\delta H$, with both $H_{{\rm PSSW},j}^{\rm YIG}$ and δH much smaller than the Py-YIG resonance distance, $\Delta H_{\rm res} = H_{{\rm PSSW},j}^{\rm YIG} - H_{\rm FMR}^{\rm Py}$. Therefore:

$$\begin{aligned} V_{\rm O} &\approx \frac{Ae^{i(\phi_{\rm L}-\phi_{\rm m})}}{(H_{\rm PSSW}^{\rm YIG}-H_{\rm FMR}^{\rm Py})-i\Delta H_{\rm Py}-\frac{g^2}{\delta H - i\Delta H_{\rm YIG}}} \\ &\approx Ae^{i(\phi_{\rm L}-\phi_{\rm m})}\frac{\frac{[\delta H - i\Delta H_{\rm YIG}]}{[H_{\rm PSSW}^{\rm YIG}-H_{\rm FMR}^{\rm Py}]}}{[\delta H - g^2\frac{[H_{\rm PSSW}^{\rm YIG}-H_{\rm FMR}^{\rm Py}]}{[H_{\rm PSSW}^{\rm YIG}-H_{\rm FMR}^{\rm Py}]^2+[\Delta H_{\rm Py}]^2}]-i[\Delta H_{\rm YIG}+g^2\frac{[\Delta H_{\rm Py}]}{[H_{\rm PSSW}^{\rm YIG}-H_{\rm FMR}^{\rm Py}]^2+[\Delta H_{\rm Py}]^2}]} \\ &\approx Ae^{i(\phi_{\rm L}-\phi_{\rm m})}\frac{\frac{[\delta H - i\Delta H_{\rm YIG}]}{[\Delta H_{\rm res}]}}{[\delta H - g^2\frac{[\Delta H_{\rm res}]}{[\Delta H_{\rm res}]^2+[\Delta H_{\rm Py}]^2}]-i[\Delta H_{\rm YIG}+g^2\frac{[\Delta H_{\rm Py}]}{[\Delta H_{\rm res}]^2+[\Delta H_{\rm Py}]^2}]}. \end{aligned} \tag{S-3}$$

where the δH is the resonance field shift of the YIG PSSW modes. As seen from Eq. S-3, the magnon-magnon coupling, characterized by the g parameter, induce an additional modulation for

both the resonance field, $\delta H(g)$, and the YIG-linewidth, $\Delta H_{\rm YIG}(g)$:

$$\begin{aligned}\delta H(g) &= \delta H - g^2\frac{[H^{\rm YIG}_{\rm PSSW} - H^{\rm Py}_{\rm FMR}]}{[H^{\rm YIG}_{\rm PSSW} - H^{\rm Py}_{\rm FMR}]^2 + [\Delta H_{\rm Py}]^2} = \delta H - g^2\frac{[\Delta H_{\rm res}]}{[\Delta H_{\rm res}]^2 + [\Delta H_{\rm Py}]^2},\\ \Delta H_{\rm YIG}(g) &= \Delta H_{\rm YIG} + g^2\frac{[\Delta H_{\rm Py}]}{[H^{\rm YIG}_{\rm PSSW} - H^{\rm Py}_{\rm FMR}]^2 + [\Delta H_{\rm Py}]^2} = \Delta H_{\rm YIG} + g^2\frac{[\Delta H_{\rm Py}]}{[\Delta H_{\rm res}]^2 + [\Delta H_{\rm Py}]^2}.\end{aligned} \tag{S-4}$$

Therefore, the YIG linewidth is broadened by the coupling coefficient, g^2 , multiplied by a Lorentzian function of $\Delta_{\rm Py}$, and the maximum linewidth of the YIG PSSW modes should be $\Delta H_{\rm YIG} + g^2/\Delta H_{\rm Py}$. The result suggests that the linewidth broadening can be enhanced by either increase the g or decrease the $\Delta H_{\rm Py}$. The corresponding data plotting and fitting are shown in the main text, Fig. 3. Finally, the spin pumping effect and antidamping torque, observed earlier in much thinner YIGs ($\sim$ 100-nm), plays a negligible role here for thicker YIG (3-μm) samples since the torque decays with the square root of the YIG thickness [6].

7. YIG FMR analysis:

Figure S-6 shows the analysis of the YIG FMR properties of the 30-nm-Py sample. A M_s value of YIG $\sim$ 0.199 T is obtained from the resonance field, Fig. S-6(c), using the Kittel formula. This value is in good agreement with that obtained from the PSSW analysis in the main text. Fig.S-6(d) plots the phase evolution with the frequency. Linear fits with the phase evolution, following $\Delta\phi_{\rm opt/ele} = \frac{2\pi f}{c}\Delta L_{\rm opt/ele}$, yield a optical/electrical path difference, $\Delta L_{\rm opt/ele} \sim$ 77.8 cm.

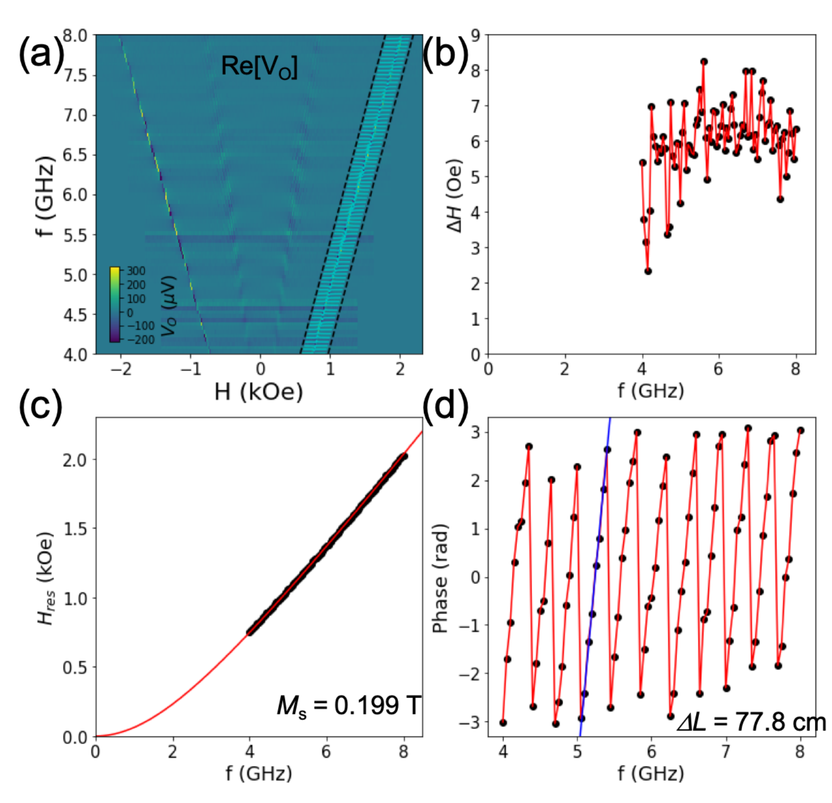

FIG. S-6. (a) The optically detected in-phase, Re[$V_{\rm O}$], for the 30-nm-Py sample, with an analysis window shifted towards the YIG FMR dispersion regime (the dashed-line pair indicates the fitting window). Summary of the (b) linewidth, (c) resonance field, $H_{\rm res}$ and the corresponding Kittel fits, and (d) the phase evolution with the frequency, are presented.

[1] S. Yoon, J. Liu, and R. D. McMichael, “Phase-resolved ferromagnetic resonance using a heterodyne detection method”, Phys. Rev. B **93**, 144423 (2016).

[2] Y. Li, H. Saglam, Z. Zhang, R. Bidthanapally, Y. Xiong, J. E. Pearson, V. Novosad, H. Qu, G. Srinivasan, A. Hoffmann, and W. Zhang, “Simultaneous Optical and Electrical Spin-Torque Magnetometry with Phase-Sensitive Detection of Spin Precession”, Phys. Rev. Applied **11**, 034047 (2019).

[3] S. Klingler, V. Amin, S. Geprags, K. Ganzhorn, H. Maier-Flaig, M. Althammer, H. Huebl, R. Gross, R. D. McMichael, M. D. Stiles, S. T. B. Goennenwein, and M. Weiler, “Spin-Torque Excitation of Perpendicular Standing Spin Waves in Coupled YIG/Co Heterostructures”, Phys. Rev. Lett. **120**, 127201 (2018).

[4] J. Chen, C. Liu, T. Liu, Y. Xiao, K. Xia, G. E. W. Bauer, M. Wu, and H. Yu, “Strong Interlayer Magnon-Magnon Coupling in Magnetic Metal-Insulator Hybrid Nanostructures”, Phys. Rev. Lett. **120**, 217202 (2018).

[5] H. Qin, S. J. Hamalainen, and S. van Dijken, “Exchange-torque-induced excitation of perpendicular standing spin waves in nanometer-thick YIG films”, Sci. Rep. **8**, 5755 (2018).

[6] Y. Li, W. Cao, V. P. Amin, Z. Zhang, J. Gibbons, J. Sklenar, J. Pearson, P. M. Haney, M. D. Stiles, W. E. Bailey, V. Novosad, A. Hoffmann, and W. Zhang, “Coherent spin pumping in a strongly coupled magnon-magnon hybrid system”, Phys. Rev. Lett. **124**, 117202 (2020).

[7] L. Liensberger, L. Flacke, D. Rogerson, M. Althammer, R. Gross, and M. Weiler, “Spin-Wave Propagation in Metallic Co25Fe75 Films Determined by Microfocused Frequency-Resolved Magneto-Optic Kerr Effect”, IEEE Magn. Lett. **10**, 5503905 (2019).

[8] H. Huebl, C. W. Zollitsch, J. Lotze, F. Hocke, M. Greifenstein, A. Marx, R. Gross, and S. T. B. Goennenwein, “High Cooperativity in Coupled Microwave Resonator Ferrimagnetic Insulator Hybrids”, Phys. Rev. Lett. **111**, 127003 (2013).

[9] X. Zhang, C.-L. Zou, L. Jiang, and H. X. Tang, “Strongly Coupled Magnons and Cavity Microwave Photons”, Phys. Rev. Lett. **113**, 156401 (2014).

[10] Y. Tabuchi, S. Ishino, T. Ishikawa, R. Yamazaki, K. Usami, and Y. Nakamura, “Hybridizing Ferromagnetic Magnons and Microwave Photons in the Quantum Limit”, Phys. Rev. Lett. **113**, 083603 (2014).
